# The Role of Sugar Signaling in Regulating Plant Fatty Acid Synthesis

**DOI:** 10.3389/fpls.2021.643843

**Published:** 2021-03-22

**Authors:** Zhiyang Zhai, Jantana Keereetaweep, Hui Liu, Changcheng Xu, John Shanklin

**Affiliations:** Biology Department, Brookhaven National Laboratory, Upton, NY, United States

**Keywords:** sugar signaling, fatty acid synthesis, metabolic regulation, SnRK1 kinase, WRINKLED1

## Abstract

Photosynthates such as glucose, sucrose, and some of their derivatives play dual roles as metabolic intermediates and signaling molecules that influence plant cell metabolism. Such sugars provide substrates for *de novo* fatty acid (FA) biosynthesis. However, compared with the well-defined examples of sugar signaling in starch and anthocyanin synthesis, until recently relatively little was known about the role of signaling in regulating FA and lipid biosynthesis. Recent research progress shows that trehalose 6-phosphate and 2-oxoglutarate (2-OG) play direct signaling roles in the regulation of FA biosynthesis by modulating transcription factor stability and enzymatic activities involved in FA biosynthesis. Specifically, mechanistic links between sucrose non-fermenting−1–related protein kinase 1 (SnRK1)–mediated trehalose 6-phosphate (T6P) sensing and its regulation by phosphorylation of WRI1 stability, diacylglycerol acyltransferase 1 (DGAT1) enzyme activity, and of 2-OG–mediated relief of inhibition of acetyl-CoA carboxylase (ACCase) activity by protein PII are exemplified in detail in this review.

## Introduction

Sugars occupy a central role in plant metabolism, acting as both metabolic substrates and signaling molecules. Well-studied cases of sugar signaling in metabolism include sucrose (Suc) promotion of fructan synthesis in grasses by the induction of fructan-synthesizing enzymes (Nagaraj et al., 2001; Noël et al., 2001). Suc also appears to act as a signaling molecule that activates starch synthesis by upregulating the expression of multiple genes involved in starch synthesis, such as the large subunit of ADP-Glc pyrophosphorylase (AGPase), granule bound starch synthase 1 (GBSS1), and β-amylase (Nakamura et al., 1991; Harn et al., 2000; Wang et al., 2001; Nagata et al., 2012) and activating the AGPase enzyme by posttranslational redox modification (Tiessen et al., 2002). Another well-known case of Suc regulation is the induction of the biosynthesis of anthocyanins. Solfanelli et al. (2006) demonstrated in experiments with *Arabidopsis* that most of the genes coding for enzymes related to anthocyanins and flavonoid syntheses are induced by Suc feeding. Emerging evidence also supports a role for trehalose 6-phosphate (T6P) as both a signal (Lunn et al., 2006) and regulator of sucrose availability (Yadav et al., 2014; Figueroa and Lunn, 2016), providing a crucial link between energy (carbon) status and the processes of growth and development (Schluepmann et al., 2003). *In vivo* T6P levels have been reported to vary over a substantial range of between approximately 7 μM in *Arabidopsis* rosettes (Martins et al., 2013) to approximately 47 μM in maize floret tissue (Nuccio et al., 2015). T6P synthase catalyzes the synthesis of T6P from UDP-glucose and glucose 6-phosphate (G6P), two activated forms of glucose (Cabib and Leloir, 1958). At the molecular level, T6P affects starch synthesis *via* posttranslational redox activation of ADP pyrophosphorylase (Kolbe et al., 2005). It was also shown that sucrose non-fermenting−1–related protein kinase 1 (SnRK1) activity in crude extracts from developing *Arabidopsis thaliana* tissues is inhibited by T6P, and this inhibition appears to depend on unknown protein factor(s) that are present only in young tissues (Zhang et al., 2009; Martínez-Barajas et al., 2011). Subsequent work showed that at much higher concentrations, G6P and glucose-1-phosphate can also inhibit SnRK1 (Nunes et al., 2013).

As an important sensor of low-carbon/low-energy status in the cell, SnRK1 is the plant ortholog of the evolutionarily conserved protein kinase family that includes the yeast sucrose non-fermenting kinase 1 (SNF1) and mammalian AMP-activated protein kinase (AMPK) (Broeckx et al., 2016). In response to cellular energy and/or carbon deficits, SNF1/AMPK phosphorylates multiple target proteins, leading to activation of catabolic processes and inhibition of anabolic processes, thereby rebalancing the energy and/or carbon status of the cell. At the molecular level, SnRK1 has been shown to phosphorylate and inactivate hydroxymethylglutaryl-CoA reductase, nitrate reductase, sucrose-phosphate synthase, and 6-phosphofructo-2-kinase/fructose-2,6-bisphosphate 2-phosphatase, which catalyze regulated steps in isoprenoid biosynthesis, nitrogen assimilation, sucrose biosynthesis, and the regulation of photosynthetic carbon partitioning, respectively (Sugden et al., 1999b; Kulma et al., 2004; Robertlee et al., 2017). SnRK1 also phosphorylates transcription factors, especially members of the bZIP family, including bZIP63, which is a key regulator of the energy/carbon starvation response in plants. The phosphorylation of bZIP63 by SnRK1 increases its ability to bind to other bZIP proteins and leads to changes in gene expression (Mair et al., 2015). SnRK1 was shown to phosphorylate B3-domain transcription factor FUSCA3 (FUS3) and increase its stability (Tsai and Gazzarrini, 2012). Accumulated evidence supports the crucial roles for SnRK1 in both normal development and responses to a wide variety of stresses that limit carbon supply, including sugar depletion, salt and osmotic stress, hypoxia, herbivory, and viral infection (Baena-González and Sheen, 2008). Structurally, both SNF1 and AMPK function as heterotrimers comprising a catalytic α-subunit together with regulatory β and γ subunits (Hardie, 2007). Although plants possess canonical α, β, and γ subunits, some of their β and γ subunits have domain architectures that are unique to plants, and a chimeric βγ subunit is the predominant γ-type subunit in SnRK1 heterotrimers (Ramon et al., 2013), suggesting that SnRK1 is a somewhat atypical member of the AMPK/SNF1/SnRK1 family (Emanuelle et al., 2015). The catalytic kinase activity of this family resides in the α subunits (i.e., KIN10, KIN11, and SnRK1.3 in *Arabidopsis*) and is strongly influenced by reversible phosphorylation of a specific threonine residue in the T-loop (also known as the activation loop) of these subunits (Estruch et al., 1992; Hawley et al., 1996; Shen et al., 2009). The degree of T-loop phosphorylation is inversely related to the energy or carbon status of the cell, because it is activated by phosphorylation under low-energy or low-carbon conditions and inactivated by dephosphorylation when energy or carbon levels increase. In mammals, the Ca^2+^/calmodulin-dependent protein kinase kinase and the serine-threonine liver kinase B1 phosphorylate T172, whereas the PP2C and PP1 protein phosphophatases are responsible for its dephosphorylation (Davies et al., 1995; Woods et al., 2005; Garcia-Haro et al., 2010). In plants, geminivirus Rep-interacting kinases GRIK1 and GRIK2 phosphorylate T175 in the T-loop of KIN10 (Shen et al., 2009; Glab et al., 2017), whereas protein phosphatases abscisic acid insensitive 1 (type 2C protein phosphatase) and type 2C protein phosphatase A mediate its dephosphorylation (Rodrigues et al., 2013). In plants, activated SnRK1 can phosphorylate and moderate the activity of GRIK, providing potential feedback control over SnRK1 activation (Crozet et al., 2010). In mammals, the binding of AMP to the AMPK γ subunit induces conformational changes that promote the phosphorylation of T172 and inhibit its dephosphorylation (Sanders et al., 2007; Oakhill et al., 2011; Xiao et al., 2011; Gowans et al., 2013). In plants, there have been fragmentary reports of AMP inhibition of SnRK1, e.g., Sugden et al. (1999a). However, a more recent study disputed these findings, based on evidence that AMP neither directly activates recombinant SnRK1 heterotrimers nor inhibits their dephosphorylation by PP2C, indicating that plant SnRK1 is not directly affected by AMP (Emanuelle et al., 2015).

Lipids are primary metabolites in cells, playing important roles as structural components of cell membranes, storing energy in the form of triacylglycerols (TAGs) and in cell signaling. Fatty acids (FAs) are major components of lipids. TAGs are mostly sequestered in lipid droplets, also known as oil bodies. Catalyzed by FA synthase, the sugar-derived substrate acetyl-CoA, along with ATP and reductant in the form of NADPH, is required for *de novo* FA synthesis. Diacylglycerol acyltransferase 1 (DGAT1) is responsible for the final step of TAG synthesis by catalyzing the conversion of diacylglycerol and fatty acyl CoAs to TAG (Bhatt-Wessel et al., 2018). Although some studies showed Suc potentiates FA synthesis, knowledge of its precise role in this process has lagged behind our understanding of the role of sugar signaling in anthocyanin and starch syntheses. Recent studies have begun to elucidate the roles of sugar signaling in regulating lipid metabolism.

## Sugar Stabilizes WRI1 and Potentiates Fatty Acid and TAG Accumulation

Lipids are derived from sugar (photosynthates), so it would be expected that higher sugar level would favor increased lipid synthesis. Sanjaya et al. (2011) observed both a 3-fold increase in Suc and 30% more TAG accumulation in leaves of AGPase-deficient plants in which *ADG1* (the small subunit of AGPase) expression was reduced by RNAi. It was also shown that *Arabidopsis* accumulated a 4-fold increase in TAG in roots when cultured on half-strength MS medium supplemented with 5% Suc than when cultured in the absence of exogenous Suc (Kelly et al., 2013). To test the influence of endogenous sugar content on FA and TAG accumulation, a high-leaf-sugar mutant was generated by reducing sugar phloem loading and starch synthesis by crossing the *suc2* (encoding a Suc/H^+^ symporter that loads Suc into phloem) mutant (Srivastava et al., 2009) with the *adg1* mutant. The sugar content (combined Glc and Suc) in *adg1suc2* leaves is 80-fold higher than that of wild-type (WT). Leaf total FA content in *adg1suc2* was 8.3% of dry weight (DW), i.e., 1.8-fold higher than WT. Leaf TAG of *adg1suc2* accumulated to 1% (DW), which is more than 10-fold higher than that of WT plants (Zhai et al., 2017b). Together, these studies confirmed that sugar potentiates TAG accumulation, lending support to the notion that sugars play roles beyond providing carbon skeletons for FA synthesis. Previous studies suggested that sugars regulate the expression of *WRI1*, an APETALA2 (AP2) transcriptional factor that induces the expression of more than 20 genes involved in glycolysis and FA synthesis (Cernac and Benning, 2004; Maeo et al., 2009). For instance, it was shown that glucose and fructose are necessary for elevated TAG accumulation in seedlings ectopically overexpressing *WRI1* (Cernac and Benning, 2004) and that the expression of *WRI1* is enhanced by Suc in *Arabidopsis* leaves (Masaki et al., 2005). Sanjaya et al. (2011) also observed that the expression of *WRI1* in seedling of AGP-deficient lines was increased compared to WT. However, later reports did not observe a significant increase in *WRI1* expression in *suc2adg1*, but a significant increase in the accumulation of WRI1 polypeptide in *suc2adg1* was observed relative to WT (Zhai et al., 2017b). Sugar-dependent regulation of gene expression and increased stability of the WRI1 polypeptide are consistent with the previously reported involvement of sugar signaling in regulating FA and TAG synthesis (Sanjaya et al., 2011).

## Kin10 Directly Phosphorylates WRI1 and Results in Its Proteasomal Degradation

While screening for genes that can increase TAG accumulation in plant vegetative tissues, transient co-expression of WRI1 and KIN10, the catalytic subunit SnRK1 in tobacco, was found to strongly suppress the level or WRI1 polypeptide and abolish its stimulation of TAG accumulation (Zhai et al., 2017a). Further studies showed that KIN10 directly phosphorylates WRI1 at sites within its two AP2 DNA-binding domains, predisposing it to proteasomal degradation (Zhai et al., 2017a). This finding is consistent with previous observations showing high sugar levels in *adg1suc2* posttranscriptionally stabilizing WRI1. KIN10-dependent degradation of WRI1 provides a homeostatic mechanism that favors FA synthesis when intracellular sugar levels are elevated, and KIN10 is inhibited; conversely, FA synthesis is curtailed as sugar levels decrease, and sugar-dependent inhibition of KIN10 abates.

## DGAT1 is a Target of KIN10

SnRK1 recognition motifs were first identified in *A. thaliana* DGAT1 (Zou et al., 1999) and subsequently in other plant DGAT1 sequences. Xu et al. (2008) demonstrated that substitution of Ser197 for Ala, within a SnRK1 recognition site in the *Tropaeolum majus* DGAT1, resulted in an increase in activity between 38 and 80%. More recently, *Brassica napus* DGAT1 was shown to be a direct substrate of SnRK1, which catalyzed its phosphorylation and converted it to a less active form (Caldo et al., 2018). SnRK1 (KIN10)–mediated modulation of DGAT1, the activity of which can limit TAG assembly (Sharma et al., 2008), provides another direct connection between cellular carbon (energy) status, sugar signaling, and TAG accumulation.

## Trehalose-6-Phosphate Inhibits the Plant Carbon/Energy Sensor SnRK1 Positively Regulating Fatty Acid Synthesis

**FIGURE 1:**
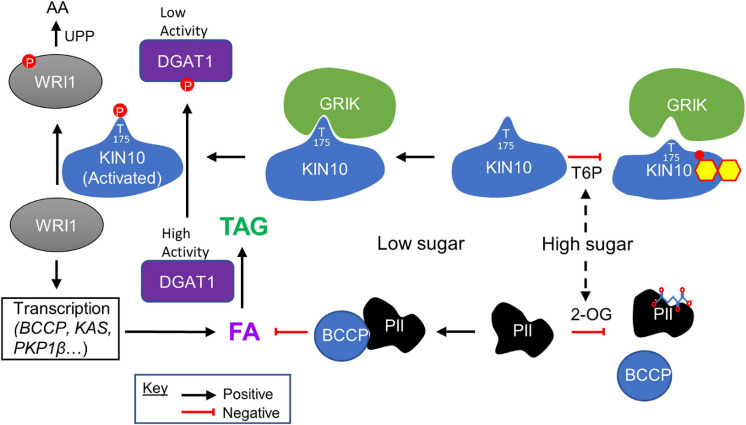
Sugar signaling in regulating plant fatty acid and TAG biosynthesis. High levels of cellular sugars (carbon) are associated with the accumulation of trehalose 6-phosphate (T6P), which binds to KIN10 (shown as small molecule superimposed on KIN10), the catalytic subunit of SnRK1, weakening its affinity for GRIK. This results in decreased phosphorylation of the KIN10 activation loop, thereby reducing the proportion of activated KIN10. Under low KIN10 activity, WRI1 is more stable and activates the transcription of genes involved in glycolysis and fatty acid (FA) synthesis such as *BCCP*, *KAS* (*3-ketoacyl-acyl carrier protein synthase*), and *PKP1*β (*plastidial pyruvate kinase 1 beta*), promoting FA biosynthesis. DGAT1 is also more active and catalyzes the conversion of diacylglycerol and fatty acyl CoA to triacylglycerol (TAG). Conversely, when the cellular sugar levels are low, levels of T6P decrease, and GRIK binds tightly to KIN10, phosphorylating its activation loop and increasing SnRK1 activity. Activated KIN10 phosphorylates WRI1 and causes its degradation *via* the ubiquitin-proteasomal pathway (UPP) to amino acids (AA), thereby reducing FA synthesis. Activated KIN10 also phosphorylates DGAT1, inhibiting its enzyme activity, reducing TAG synthesis. High levels of cellular sugars are also associated with the accumulation of TCA cycle intermediates including 2-OG, which binds to PII protein (shown as small molecule superimposed on PII), disrupting its interaction with BCCP. This blocks the ability of PII to inhibit ACCase activity, thereby promoting FA biosynthesis. Conversely, when the cellular carbon level is low, the level of 2-OG decreases, and PII binds tightly to BCCP inhibiting ACCase activity and consequently FA biosynthesis.

That WRI1 is a target of KIN10 that suggested T6P, a potent inhibitor of KIN10 (Zhang et al., 2009), might stabilize WRI1 and positively regulate FA and synthesis and TAG assembly. In a recent study employing microscale thermophoresis, a technique for directly measuring dissociation constants (K*d*s), T6P was demonstrated to directly bind to KIN10 at physiologically relevant concentrations. The same approach was used to demonstrate that T6P binding weakens the KIN10-GRIK1 association, thereby reducing the activation of KIN10 and thus SnRK1 activity (Figure 1). T6P inhibition of KIN10 activity was shown to be strictly GRIK-dependent, in experiments using either purified recombinant proteins or crude *Arabidopsis* leaf extracts. Extracts from young leaves of *grik1*, *grik2*, and *grik1grik2* mutants showed reduced inhibition of KIN10 relative to those derived from WT leaves, confirming the dependency of T6P-mediated inhibition of KIN10 on GRIK1 (Zhai et al., 2018).

## 2-Oxoglutarate Binding to PII Regulates Acetyl-CoA Carboxylase

Another example of sugar signaling directly involved in regulating FA synthesis is through the PII protein. The PII protein is a signal integrator involved in the regulation of nitrogen/carbon homeostasis in bacteria (Gerhardt et al., 2015) and plants (Uhrig et al., 2009). PII binds ATP and 2-oxoglutarate (2-OG), and depending on the degree of ligand binding, it interacts with a constellation of enzymes, transcription factors, and transporters, modifying their activities (Arcondéguy et al., 2001; Ninfa and Jiang, 2005; Osanai and Tanaka, 2007; Forchhammer, 2008; Uhrig et al., 2009). In plant plastids, PII binds to biotin carboxyl carrier protein of acetyl-CoA carboxylase (BCCP), a subunit of the plastidial heteromeric acetyl-CoA carboxylase (ACCase), and inhibits ACCase activity in chloroplast extracts by up to 50%. The tricarboxylic acid (TCA) cycle intermediates 2-OG, pyruvate and oxaloacetate (OAA) were shown to completely reverse this PII-dependent inhibition of ACCase (Bourrellier et al., 2010) (Figure 1). The sugar derivatives OAA and pyruvate are not only involved in acetyl-CoA synthesis but are also closely related to the cellular carbon/energy status. It was shown by Nukarinen et al. (2016) that sugars are lower, whereas TCA intermediates including 2-OG, OAA, and pyruvate are elevated, in the *snrk1*α*1*/α*2* mutant under extended dark conditions. By binding with TCA intermediates, PII conveys the signal of cellular carbon status directly to ACCase, thereby regulating FA synthesis.

## Summary and Future Perspectives

Recent reports elucidating the mechanisms by which 2-OG suppresses PII-mediated ACCase inhibition and T6P disrupts SnRK1-mediated turnover of WRI1 along with modulation of DGAT1 activity are revealing the critical roles of sugar signaling in lipid synthesis. It is interesting to note that while PII and KIN10 are unrelated evolutionarily, both mechanistically act as metabolic sensors that bind 2-OG and T6P, respectively. Further, the metabolite binding reduces their affinity for their respective protein binding partners, in both cases making FA synthesis contingent upon the availability of sufficient metabolic resources to support the process. While the research summarized herein has begun to fill a key knowledge gap in metabolic regulation by establishing clear mechanistic connections between sugar signaling and lipid synthesis and accumulation, it is anticipated that many additional connections will be discovered in the near future. Potential connections are included but not limited to the yet-to-be identified E3 ubiquitin ligase of WRI1 or other key enzymes of lipid metabolism that may be involved in sugar signaling, unknown regulators of WRI1 or LEC2 transcription that may also be a part of the carbon sensing network. Sugar signaling may also play roles in oil body stability and regulation of fatty acid degradation in peroxisomes from the perspective of carbon(energy) demand.

## Author Contributions

ZZ drafted and edited text and figure. JK and HL drafted and edited text. JS conceived the work, contributed to draft, and edited both text and figure. CX edited text. All authors contributed to the article and approved the submitted version.

## Conflict of Interest

The authors declare that the research was conducted in the absence of any commercial or financial relationships that could be construed as a potential conflict of interest.

## Glossary

**Table:**

T6P	Trehalose 6-phosphate
2-OG	2-Oxoglutarate
SNF1	Non-fermenting kinase 1
AMPK	AMP-activated protein kinase
SnRK1	Sucrose non-fermenting-1–related protein kinase1
GRIK	Geminivirus Rep-interacting kinase
TAG	Triacylglycerol
DGAT1	Diacylglycerol acyltransferase
ADG1	Small subunit of ADP-Glc pyrophosphorylase
ACCase	Acetyl-CoA carboxylase
BCCP	Biotin carboxyl carrier protein of acetyl-CoA carboxylase
KAS	3-Ketoacyl-acyl carrier protein synthase
PKP1β	Plastidial pyruvate kinase 1 beta
